# Open Biomedical Ontology-based Medline exploration

**DOI:** 10.1186/1471-2105-10-S5-S6

**Published:** 2009-05-06

**Authors:** Weijian Xuan, Manhong Dai, Barbara Mirel, Jean Song, Brian Athey, Stanley J Watson, Fan Meng

**Affiliations:** 1 Psychiatry Department and Molecular and Behavioral Neuroscience Institute, University of Michigan, USA; 2 School of Education, University of Michigan, USA; 3 Health Science Libraries, University of Michigan, USA; 4 National Center for Integrative Biomedical Informatics, University of Michigan, USA

## Abstract

**Background:**

Effective Medline database exploration is critical for the understanding of high throughput experimental results and the development of novel hypotheses about the mechanisms underlying the targeted biological processes. While existing solutions enhance Medline exploration through different approaches such as document clustering, network presentations of underlying conceptual relationships and the mapping of search results to MeSH and Gene Ontology trees, we believe the use of multiple ontologies from the Open Biomedical Ontology can greatly help researchers to explore literature from different perspectives as well as to quickly locate the most relevant Medline records for further investigation.

**Results:**

We developed an ontology-based interactive Medline exploration solution called PubOnto to enable the interactive exploration and filtering of search results through the use of multiple ontologies from the OBO foundry. The PubOnto program is a rich internet application based on the FLEX platform. It contains a number of interactive tools, visualization capabilities, an open service architecture, and a customizable user interface. It is freely accessible at: .

## Background

The popularity of data driven biomedical research leads to large volumes of data such as gene expression profiles, MRI images and SNPs related to various pathophysiological processes. As a result, understanding the biological implications of high throughput data has become a major challenge [1]. It requires time-consuming literature and database mining and is the main goal of "literature-based discovery," "conceptual biology," or more broadly, "electronic biology," which aims at deriving biologically important hypotheses from existing literature and data using *in silico *approaches [2-9]. The effectiveness of such knowledge mining also relies heavily on researchers' background knowledge about the related data such as novel genes or SNPs, and this knowledge, at present, is sparse.

The Medline database is without doubt the foremost biomedical knowledge database for understanding high throughput data. However, one major shortcoming in prevailing Medline search engines such as PubMed and Google Scholar is that they are designed largely for the efficient retrieval of a small number of records rather than for an in-depth exploration of a large body of literature for discovery and proof purposes. They rely heavily on a step-wise narrowing of search scope [10,11] but such an approach does not work well for the exploration of uncharted territories. In exploration, researchers must be able to apply their background knowledge to define sensible filtering criteria and to infer potentially relevant topics from query results for additional exploration. For example, in microarray gene expression analysis, researchers frequently have to deal with lists of genes that are not known to be associated with the targeted biological processes. Researchers have to utilize other intermediate concepts to establish indirect links between gene lists and specific biological processes. However, identifying such intermediate concepts is very difficult in existing solutions [12], and it is not easy even in systems devoted to this purpose such as ArrowSmith [13,14]. Frequently researchers have to go through large numbers of retrieved records one-by-one and examine external databases to find interesting new relationships. As bioinformatics specialists note, "Continued work is needed to enhance these systems to handle the vast amounts of different types of data that scientists currently must explore manually" [15].

Another shortcoming for prevailing search solutions is that they do not present results in contexts that a user may be interested in. For example, Google Scholar/PubMed presents search results as a linear list of papers, arranged according to citation rate or publication date. Users do not know the context of each paper nor the explicit relationship among the papers. Besides the displayed ranking provided by the search engine, there are few additional cues and sorting/filtering methods that can facilitate the exploration of search results.

We believe the projection of search results to existing knowledge structures is very important for hypothesis development. Cognitive research on scientists' ways of knowing and reasoning shows that to formulate hypotheses from displayed data scientists require cues – data arrangements and conceptual indicators that direct them toward drawing accurate and relevant inferences [16]. These cues determine whether researchers can simply read off lists or can more deeply place retrieved items in a biological context and story [17]. Even if a researcher wants to examine and read off facts in his or her own field from data displays, there are many details related to the search topic that will still require additional efforts to retrieve, and many of these are not easily identified by prevailing approaches. For example, if Medline search results show that several genes in a brain region are related to a disease in a statistically significant manner, it would be worthwhile to explore the relationship of other genes expressed in this brain region with the disease. Exploring such multiple knowledge structures is often needed to facilitate the formation of new insights. Thus ideally, projecting search results to multiple dynamically-linked knowledge structures could provide the context-assisted data and literature exploration needed for new insights.

Some newer Medline search/mining solutions such as GoPubMed [18], Vivisimo [19], Textpresso [20], and OBIIE [21] attempt to organize search results in the context of either a predefined ontology such as Gene Ontology [22] or dynamically generated ontology structures based on clustering results. Such solutions present a tree-like organization of search results to help users easily navigate to topics of interest. Moreover, the neighbourhood of a given tree branch automatically suggests related topics for additional exploration. Here predefined ontologies have an advantage over clustering results for exploring unfamiliar territories due to their systematic listing and thus cuing of related concepts and their relationships.

These approaches rely on only one or two ontologies for effective exploration, still leaving out or failing to adequately cue many potentially relevant articles previously unknown to the researcher. There are huge numbers of biomedical concepts (e.g., over 1 million in the Unified Medical Language System), and the relationships among them that can be associated with articles are complex. In keeping with scientists' higher order reasoning for exploratory analysis, researchers need to have better tools for formulating searches and examining search results from these different perspectives, including conceptual cues to guide them toward finding biologically meaningful hits. We believe an ontology-based Medline exploration solution must allow the use of different orthogonal ontologies, i.e. ontologies that address different aspects of biomedical research [23]. In addition, for more efficient Medline exploration and for exploration grounded in researchers' domain expertise, it is critical to enable interactive filtering of search results using terms from different ontologies.

The main goal of this work is to develop a flexible ontology-based Medline exploration solution to facilitate the understanding of high throughput data. The solution reveals to researchers potentially interesting conceptual relationships in studies done by other scientists in the target area of investigation. Additionally, the solution lets researchers selectively view relevant articles by conceptual category within and across multiple levels of different conceptual hierarchies (ontologies); and it lets them interact with visualized ontologies to see search results from diverse perspectives without having to write and re-write query statements. The solution uses multiple ontologies from the OBO foundry. It also has an open architecture that allows flexible selection of Medline retrieval algorithms through different web services.

## Methods

### Selection of ontologies

The Open Biomedical Ontologies (OBO) foundry is a comprehensive collaborative effort to create controlled vocabularies for shared use across different biological and medical domains [24-26]. It already includes around 50 ontologies from various biomedical domains. We selected Gene Ontology, Foundational Model of Anatomy [27], Mammalian Phenotype Ontology [28], and Environment Ontology [29] for inclusion in our prototype since they provide key perspectives for topics of great interest for many biomedical research and they are almost orthogonal to each other conceptually.

### Mapping Medline to ontology

We developed a highly efficient, general purpose ontology to free-text mapping solution in collaboration with researchers in the National Center for Biomedical Ontology. In brief, our solution relies on the pre-generation of lexical variations, word order permutations for ontology terms, their synonyms together with a highly efficient implementation of a suffix-tree based string match algorithm. Our solution is able to map all concepts in UMLS to the full Medline database in 15 hours on a mainstream Opteron server. It achieves over 95% recall rate for UMLS terms when compared to the results from the MMTx program [30], which does not support the use of non-UMLS ontologies. We will describe the technical details and systematic evaluation of our ontology mapping solution in a separate manuscript. Our engine is currently available for web-based OBO annotation for biomedical text at .

### PubOnto architecture

**Figure 1:**
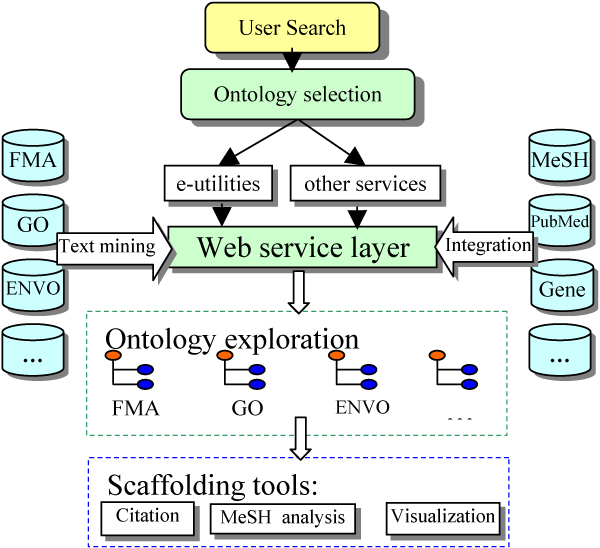
**The component-based and open architecture of PubOnto**. Technologically, PubOnto is developed based on Adobe's latest Flex 3.0 platform. It allows us to build a highly interactive user interface that is compatible in virtually all major browsers.

In order to provide a web-based Medline exploration tool with rich interactivity, we developed PubOnto on Adobe's latest Flex 3.0 platform. It allows us to build a highly interactive user interface that is compatible in virtually all major browsers. We developed an innovative technique that dynamically updates the XML-based ontology tree structure by building a web service for each ontology for feeding expanded nodes with ontological information and literature searching results. As a result, only a minimum amount of data is transferred asynchronously, and PubOnto can thus handle very large ontologies. Figure 1 shows the architecture of PubOnto. Since the web service layer separates the user interface from ontologies, search services and other databases, the back end changes do not affect the client side user interface.

## Results

PubOnto is a FLEX application that provides users with a high level of interactivity for efficient Medline search result exploration. We illustrate a number of key features in this section, tying the features to a use case of exploratory querying and analysis.

### Efficient ontology traversal and aggregation

**Figure 2:**
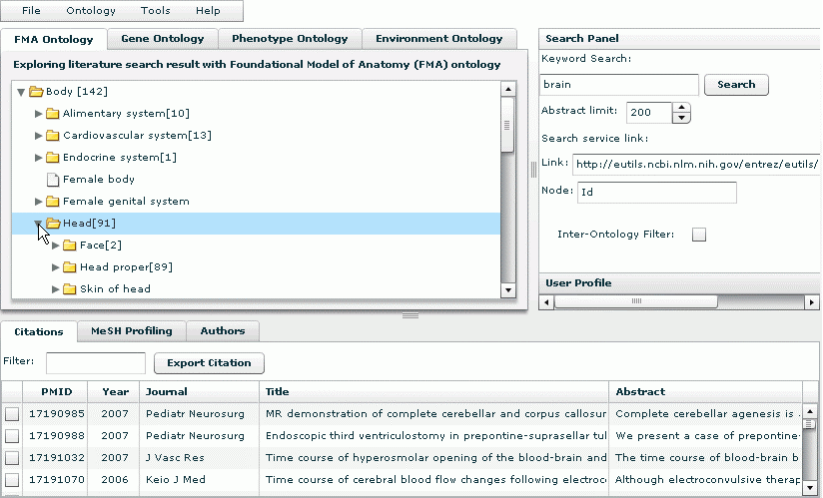
**The display of results grouped by ontological categories**. It also shows how many articles are retrieved for all children under a branch of any given ontology category.

In terms of a use case, a researcher who wants to explore the literature conceptually will enter a query term, e.g. "brain" and then examine retrieved results. He or she will want to see, generally, how retrieved articles are distributed across diverse biological concepts and will want to identify, specifically, which articles deal with the researcher's areas of interest. After submitting the query, the researcher will get a display of results grouped by ontological categories that show how many articles are associated with each superordinate node. He or she will want to know quickly how many articles are retrieved for all children under a branch of any given ontology category – a critical requirement for being able to decide on interesting aspects that need to be explored further. To see these subordinate nodes and to explore the articles associated with them, the researcher will expand the tree nodes by clicking on them, as shown in Figure 2; correspondingly, the researcher will collapse tree branches as needed. Expanding and collapsing will be quick and easy.

Technologically, detailing and rolling up such mapped data in a large ontology on-the-fly is not an easy task. Traditional tree traversal algorithms are very CPU intensive and usually require large in-memory tree structures on the server. To provide real time interactivity, we pre-traversed each ontology and generated a parent-child table that matches all nodes in the sub-tree to their parent nodes. When a user issues a query, PubOnto server-side programs save the literature retrieval results to a session-based table. When a user expands a node, our service performs an efficient table join to obtain the aggregated information. This simple and effective algorithm not only provides fast response to user queries but also eliminates the memory pressure on the servers that usually are imposed by ontology traversal and aggregation algorithms that run on-the-fly. This makes PubOnto highly scalable in supporting concurrent users running analyses on multiple ontologies.

Another benefit of this approach is that subsequent exploration of search results on the ontology will be directly returned from a simple database query without any further search, traversal and aggregation. Therefore, from the perspective of a user's experience, PubOnto is highly responsive in interactive explorations.

### Ontology-based search result exploration

Continuing with the use case, the biomedical researcher who engages in expanding and collapsing tree nodes in a given ontology will have specific intentions. First, the researcher will want to find concepts of interest and associated articles within the ontology without intervening distractions from the display modes of any other ontologies. Second, the researcher will want to scan and selectively review articles that are associated with both a conceptual category in one ontology and a conceptual category in another ontology. PubOnto features accommodate these different user needs by giving users two search mode options: an independent ontology search mode and an inter-ontology filter mode.

In the independent ontology search mode, when a user clicks the name of an individual ontology concept, the concept becomes highlighted, and citations associated with the concept and its sub-concepts in the current ontology are displayed as a list in the bottom panel (see Figure 2). PubOnto provides an interactive means for expanding parent nodes to their children – namely, users clicking on the arrow next to a concept. It also provides an interactive means for generating a list of associated citations – users clicking on the concept name itself. Numbers in brackets next to a concept name indicate a count of articles for each parent and children node.

**Figure 3:**
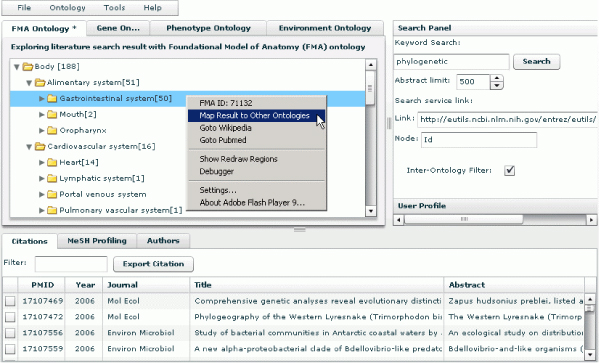
**PubOnto's inter-ontology filter mode, which shows intersecting articles between different ontologies**. In the ontology tab that is open, a user specifies a node/categorical concept of interest by clicking on its name, and then right clicks on the name of this concept, and a context menu appears. Select the "Map Result to Other Ontologies" activates PubOnto's automatic mapping of one ontological term to the terms in another ontology.

In PubOnto's inter-ontology filter mode, when a user clicks the checkbox next to "Inter-ontology filter" (in the lower portion of the Search panel), the search mode for showing intersecting articles between different ontologies is activated (see Figure 3). The inter-ontology filter mode works as follows. In the ontology tab that is open, e.g. FMA, a user specifies a node/categorical concept of interest by clicking on its name. The user then right clicks on the name of this concept, and a context menu appears. On this menu, the user selects "Map Result to Other Ontologies," as shown in Figure 3. This selection activates PubOnto's automatic mapping of one ontological term to the terms in another ontology. A user sees the results of this mapping by moving to the other ontology. In the other ontology, the count of articles in brackets after the concept name reflects articles belonging to that ontological concept and the the concept mapped from the original ontology. As in the independent search mode, a user can see associated inter-ontology citations by clicking on the name of a mapped concept.

The inter-ontology filter allows a user to easily change concept mappings. If a user returns to the original ontology and chooses a new concept for exploration, he or she can map this new concept to another ontology by selecting "Map Result to Other Ontologies" from the right-click context menu. PubOnto now cancels the previous filtering and maps this newly selected concept to the other ontologies, thereby filtering to a new set of intersecting articles across ontologies.

This novel functionality provides users both the ontological mapping overview across multiple concept spaces and the detailed results as users navigate through the ontology mapping results. It addresses an important issue that is often encountered by ontology- or clustering-based solutions, which is a lack of interoperability between orthogonal concept spaces.

### Ontology selection

**Figure 4:**
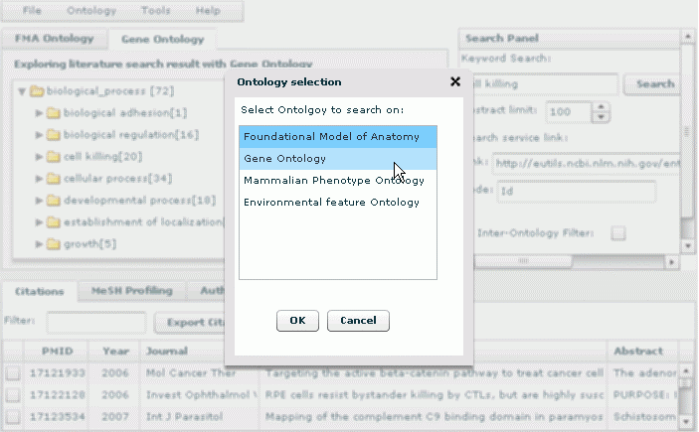
**PubOnto supports a flexible way for users to choose which of the supported ontologies they want to use**. When a user selects certain ontologies of interest, PubOnto creates a new user interface that presents only the ontologies the user selected.

Making ontology selections is also part of a typical use case. A researcher, at times, will value exploring results for all the ontologies and, at other times, will want only to see one or two of them. From a use case perspective, rather than "the more function the better," it is "the driving principle is the more suitable the better". PubOnto currently supports four aforementioned OBO ontologies, and with the algorithms and infrastructure implemented, it can easily incorporate a series of many other OBO ontologies. However, in the biomedical research community, researchers usually are specialized in sub-fields such that only a few ontologies are pertinent to them when they perform literature research. Therefore, we present a flexible way for users to choose which of the supported ontologies they want to use, as shown in Figure 4.

Most existing online literature research tools have rigid user interfaces that do not address heterogeneous user needs. Our approach is different. Ontologies supported by PubOnto are shown in the list. Once a user selects one or more ontologies of interest, PubOnto dynamically creates a new user interface that presents only thsoe ontologies. Each of the ontology tabs has consistent navigational and interactive functions. Search and interaction in PubOnto are conducted only on the selected ontologies. Therefore, this ontology selection feature also reduces the amount of computation required and enhances user interface responsiveness.

### Medline citation presentation and analysis

As the use case being presented here suggests, a biomedical researcher will conduct his or her search and analysis tasks by moving back and forth between conducting an analysis of the conceptual distribution of retrieved results and making judgments about the relevance of specific citations associated with conceptual groupings. Seeing details about specific citations will enable the researcher to glean additional information for relevance judgments, for example from the title, author, journal name, date of publication, abstract, and associated MeSH terms. Once the researcher finds articles of interest, he or she can export them for future reference.

**Figure 5:**
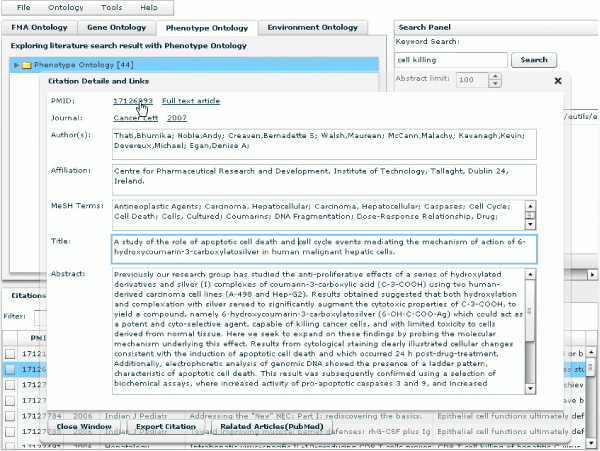
**In addition to displaying citations in table format, PubOnto can show citations in detail**. To view the detailed record, a user can click the citation to bring up a "Citation Details and Links". It shows Medline record information of this citation, along with links to external resources.

PubOnto provides users with these export capabilities and with details on citations in the bottom tabbed panel. The panel contains three tabs. The "Citations" tab presents citations associated with an ontology's node (and its children nodes) selected by a user. PubOnto enables a user to view a summary line about each citation or to view in detail the corresponding Medline record. To view the full Medline record, a user can click the citation to bring up a "Citation Details and Links" window, as shown in Figure 5. It shows Medline record information for this citation, along with links to external resources.

**Figure 6:**
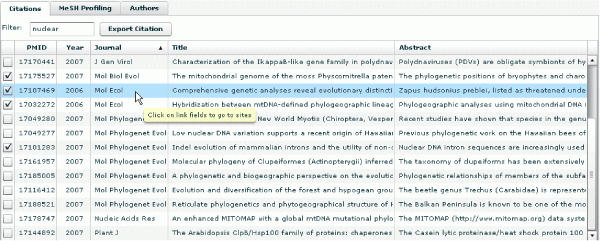
**PubOnto provides citation filtering capabilities**. To filter citations on the client side, a user can type any keywords in the filter input field, and the citations will be filtered dynamically as users type the keywords. To return to the original citation list, the user just needs to delete the filter string.

PubOnto provides citation filtering capabilities, as well. To filter citations in real time (on the client side) a user can type any keywords in the filter input field, and the citations will be filtered dynamically as users type the keywords. To return to the original citation list, the user just needs to delete the filter string, as shown in Figure 6.

Additionally, because PubOnto is designed to be a Rich Internet Application, it provides users a desktop-like experience. It provides features that enable users to sort citations by any column through a click on the column headers, to re-arrange columns through drag-and-drop header techniques, to resize panels via drag-and-drop panel splitters, and to select multiple citations to process by checking the checkboxes.

PubOnto also satisfies users' needs to export citations of interest. When users choose "Export Citation" PubOnto will format citation information, automatically launch Endnote installed on the client side, and import the information to the citation library that the user specifies.

**Figure 7:**
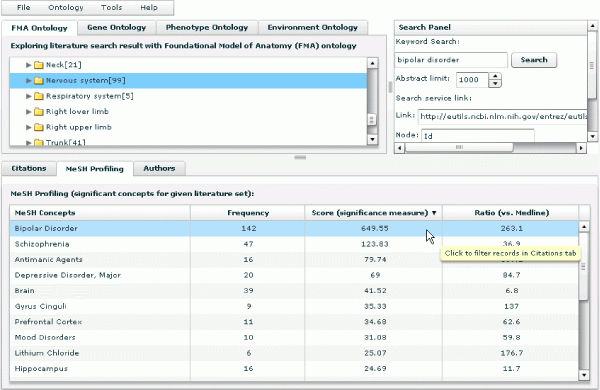
**MeSH profiling function aims to help users identify themes and contexts of the publications so that they can better identify those of interest**. It identifies the most significant MeSH concepts that differentiate the current citation group from the whole Medline corpus.

In another tab in this lower panel, "MeSH Profiling," the aim is to help users identify themes and contexts of the publications to facilitate users in exploring potentially related publications. The "MeSH Profiling" function identifies the most significant MeSH concepts that differentiate the current citation group from the whole Medline corpus. We adapted the corpus profiling algorithm [31], which ranks terms that differentiate two corpora the most. In PubOnto, we always derive the rank of terms based on the retrieved records vs. all Medline records. For example, if a user queries for "bipolar disorder" and selects "Nervous system" from the FMA ontology, the MeSH concept "Bipolar Disorder" is ranked the first by the algorithm, and other related concepts such as disorders, brain structures and drugs also rank among the top. These concepts should be very useful for researchers not familiar with this area to conduct further exploration for hypothesis development (Figure 7). Because we have pre-calculated the frequency of each MeSH term in Medline, the profiling calculation only takes less than half a second. Simple frequency counts and ratios of the frequency of a term in the current group to the whole Medline corpus are also provided so that users can sort the MeSH concepts by any of these criteria.

In the third tab, "Authors," PubOnto offers a summary of the authors of the current citation group. A user can sort by citation count to find authors published frequently on this topic or follow the links in the table to find other publications of an author. Meanwhile, with the same techniques and underlying reasoning discussed in the previous section, PubOnto allows a user to customize display of the data analysis tabs.

### Data visualization

**Figure 8:**
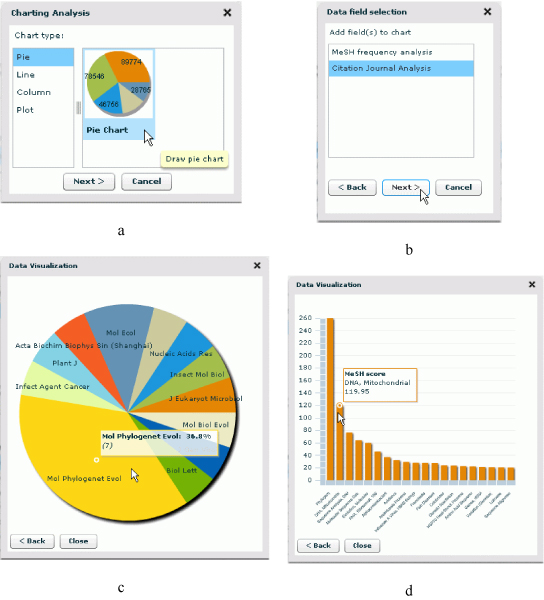
**PubOnto offers visualization tools to help users interpret the structure of retrieved results**. It supports four types of common charts, including pie chart, line, column, and plot charts. Each chart displays the data that are best suited to the analysis supported by the graph type. Figure 8a-8d demonstrates the procedures and outcomes of using these visualization tools.

Data visualizations are relevant to our exploratory search and analysis use case, as well. A biomedical researcher will want to identify quickly which ontological categories in one of more ontology contain retrieved articles. He or she also will want to conceptualize how the articles overall are distributed across these ontological categories and what trends characterize publication dates of articles in various conceptual categories. Grasping these "results profiles" will help direct the researcher to the concepts and articles most pertinent to his or her goals. We provide visual presentations to enable analysts to quickly grasp frequencies, distributions, and trends [32]. PubOnto has separate graphic tools to facilitate users in discerning from the visualized structure of retrieved results the spread of concepts relevant to the query terms or the apportioning of retrieved articles in these concepts. To graphically display frequencies, distributions, and trends PubOnto provides four types of common charts, including a pie, line, column, and plot chart (Figure 8a). Each chart displays the data suited to a given type of analysis. For example, a user can pick a pie chart to visualize the proportionate spread of journals (pie slices) in a given result set. This view immediately shows users which domain specialities devote more coverage to the topic being searched than others. See Figure 8 for the procedures and outcomes of displaying these PubOnto visualization tools.

All charts are interactive. For example, using the PubOnto mouse over function a user can view more details of a graph. A user also can go back to previous selections to change data sources or chart types (Figure 8d). Note that for some charting, PubOnto automatically performs and displays aggregations after a user specifies the chart type and data source. For other charts PubOnto uses the same data displayed in the tables. Yet visual displays present results more intuitively for interpretation.

### Search service and open architecture

Information retrieval algorithms in the biomedical fields often have pros and cons but existing Medline search solutions do not take this into consideration. We believe one search algorithm does not fit all needs in diversified biomedical fields. Thus we designed an open architecture that can incorporate other search algorithms through web services in addition to the default search service. In the default search service, when users submit a query the client-side program sends web service requests to service programs on the server. These service programs perform the following tasks: 1) transform the query and other criteria, 2) call search services provided by PubMed, 3) extract PMIDs, 4) map to pre-indexed Medline-to-ontology mapping tables, 5) aggregate results and handles potential search exceptions, and 6) finally return the results in web service format to the client program.

In PubOnto, the service is designed in a component-based architecture. Such a design enables us to open the tasks 2) and 3) above to other search services that can be specified by users (where the users also need to specify the element that contains PMID). Once the result is retrieved the PMIDs are extracted from a third-party web service and the subsequent tasks are performed as in the default mode. In either mode, the search service is controlled by our service program instead of directly interacting with the client side program. The operations are transparent to users without problems related to security or cross-domain accessibility. The only change, which is also the purpose of this architecture, is the difference in search results that reply on the search service the user specified.

### Other scaffolding tools

From a use case perspective, a researcher expects searching, creating filters, and navigating to be easy and efficient. PubOnto offers several tools for these purposes, including an auto-complete function for keyword search, customizing features in the context menu mentioned previously, and user session management.

**Figure 9:**
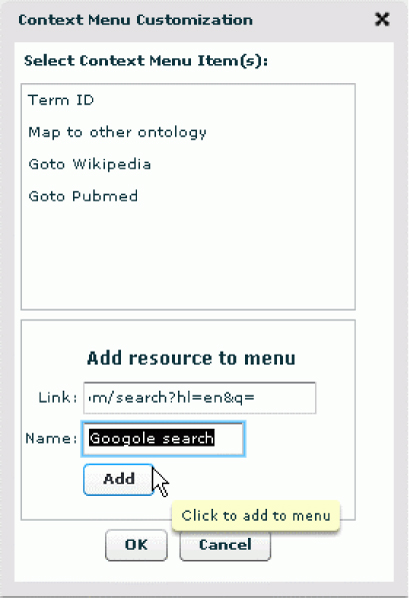
**PubOnto builds in a context-sensitive menu that can be accessed by a right click on the ontology node**. In addition to the default options on the context menu, users can add other search engines or other services. The customized context-sensitive choices behave the same way as built in options.

**Figure 10:**
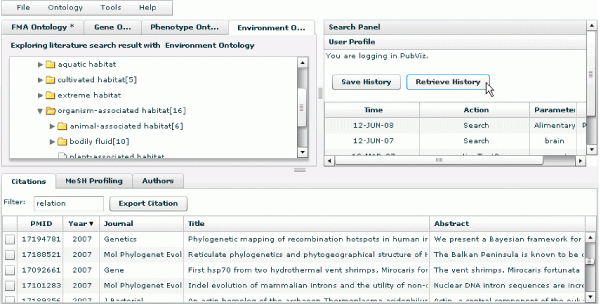
**User session management**. PubOnto prototyped a user session management function. It records users' search history and their choice of parameters. After logging in, users can save or trace their search sessions.

1) Auto-Complete function. This function facilitates typing in search keywords in the input box, thereby making users' formulations of queries efficient. As a user types, PubOnto make a service call, which matches the string to entities in ontology databases and returns a list of partially or fully matched terms. While somewhat sophisticated in implementation, it is intuitive to users (it behaves the same as other popular search engines do, e.g., Google's auto-complete function.).

2) Customizable context menu. During the ontology-based exploration, users may find literature hits pointing to concepts that are unfamiliar to them. In such situations, a user will want to quickly examine the meaning of the concept or search for the context in which the concept is used. Typically, a user will want to turn to his or her favourite search engine, and PubOnto accommodates this intention. PubOnto implements a context-sensitive menu that can be accessed by a right click on the ontology node. Existing options in the menu include getting term definitions from our in-house integrated ontology database and directing to specific entries in Wikipedia or PubMed. A user can add other search engines or other services, as shown in Figure 9. A user can also decide what menu items the context menu entry should have. The customized context-sensitive choices behave the same way as built in options.

3) User session management. The interactive exploration capability of PubOnto enables users to search and filter results in more combinations than typical tools provide but it also makes it harder to trace back the processes. PubOnto thus builds in a user session management function. It records users' search history and their choice of parameters (Figure 10) so that users can save or trace their search sessions.

In summary, while the most important feature of PubOnto is the ability to use multiple OBO ontologies for Medline exploration, it also offers a number of unique features summarized in Table 1. Table 1 compares our solution with GoPubMed, which is arguably the most popular ontology-based literature search program in the biomedical field. The PubOnto prototype currently does not include several functions that GoPubMed has, as they are not directly related to ontology, but similar functions will be added in the future upon users' request.

**Table 1:** Comparison between PubOnto and GoPubMed

	**PubOnto**	**GoPubMed**
More ontologies besides MeSH, GO	Yes	No
Interaction among ontology	Yes	No
Customizable search service	Yes	No
Client side filter	Yes	No
Customizable ontology search	Yes	No
Customizable interaction function	Yes	No
Rich interactions	Yes	No
Search history maintenance	Yes	Yes
Sorting citation by various criteria	Yes	No
Export to Citation managers	Direct	Indirect
Citation linkouts	Yes	Yes
Where/Where/When analysis	Some	Yes
Keyword highlight	No	Yes
Hot topics	No	Yes
Wikipedia mapping	Yes	Yes

## Discussion

Systematic ontology development efforts such as those related to the Open Biomedical Ontologies are likely to generate an expansive conceptual framework for the integration, analysis and understanding of data generated in different areas of biomedical research. PubOnto aims to capitalize on the impressive progress in ontology development for the exploration and mining of biomedical literature. The ability to utilize multiple orthogonal ontologies during Medline exploration can significantly increase the efficiency of locating interesting search results in areas that researchers are not familiar with. Mapping Medline results to multiple ontologies also enables researchers to explore search results from different angles for new hypothesis development.

While PubOnto enables the use of multiple ontologies for Medline exploration, there are a number of improvements we hope to incorporate in future versions. For example, although the ability to select different ontologies for organizing search results is quite powerful, it is based on the assumption that users know which ontologies they want to use. In reality, due to the quick expansion of OBO, a typical user may not know the most useful ontologies for his/her literature exploration beforehand. It will be ideal for PubOnto to provide some automated ontology selections as the starting points of exploration. We plan to develop methods to rank ontologies for their usefulness to a specific topic based on the distribution of returned Medline records on different concepts under a given ontology. For example, an ontology is not very useful for Medline search result exploration if only a small fraction of returned records can be mapped to this ontology. On the contrary, an ontology will be very effective if many records can be mapped to it and if those records are relatively evenly distributed across many terms in that ontology. Of course, an ontology will still not be useful if most of the search results can be mapped to only a few terms in an ontology. Consequently, it should be possible to develop an ontology scoring system based on the number of records that can be mapped to an ontology and the distribution of Medline records in an ontology for the automatic selection of a default ontology for a given Medline search result. Conceivably, once the first ontology is selected, it is possible to select the second best ontology based on the "orthogonality" with the first ontology. Of course, such automated ontology ranking procedures are only based on the statistical properties of the Medline records to ontology mapping. Users' biomedical knowledge and their understanding of different ontologies will be essential for effective exploration of Medline literature.

Similarly, the exploration of a given ontology tree currently is also dependent on users' background knowledge since only the number of Medline records hits for a given term can be used as external cues for ontology exploration now. If there are many different ontologies from which a user is to select or if a user is not familiar with the corresponding ontology at all, it is desirable to have additional information to help users engage ontologies for exploration more effectively and efficiently. It will be pretty straightforward to weigh the specificity of each ontology term based on their inverse frequency of showing up in the Medline corpus so that users can focus on more specific terms rather than exploring generic terms.

Another improvement involves web services. PubOnto is designed with a client-server model, and all the communications between the client and server are through standard web service or http service calls. As we have done for other programs, we will open our ontology-based search and analysis web services to the community to help the development of other research projects.

These technological advances will be coupled with user testing. As Cohen and Hersh [15] highlighted, many retrieval and analysis tools in bioinformatics fall short in supporting scientists' actual exploratory analysis. We will use formative usability testing to assure that designs of current and new features and displays adequately match the purposes and practices of scientific researchers. Testing will uncover, as well, necessary improvements for ease and efficiency of use, navigation, comprehension and satisfaction. After fitness-to-purpose and usability improvements are implemented, we will conduct comparative user performance evaluations with systems such as PubMed and GoPubMed with the aim of establishing the benefits of PubOnto's technological advances and user-centered design.

## Conclusion

We believe the use of multiple ontologies in OBO for Medline exploration can significantly increase the efficiency of Medline exploration and facilitate the examination of the same search result from different perspectives. We will continue to improve PubOnto to make it an effective tool for novel biomedical hypotheses development, and ultimately incorporate it into PubViz, our more comprehensive biomedical literature exploration engine.

## Competing interests

The authors declare that they have no competing interests.

## Authors' contributions

W. Xuan is the main developer of the PubOnto project. M. Dai developed the free text to Ontology mapping engine used in this project. All authors participated in the design, evaluation and improvement of the PubOnto with main efforts from W. Xuan, B. Mirel and F. Meng. All authors contributed to and approved the manuscript.
